# Frames of reference and categorical/coordinate spatial relations in a “what was where” task

**DOI:** 10.1007/s00221-016-4672-y

**Published:** 2016-05-14

**Authors:** Francesco Ruotolo, Tina Iachini, Gennaro Ruggiero, Ineke J. M. van der Ham, Albert Postma

**Affiliations:** 1 Helmholtz Institute, Experimental Psychology, Utrecht University, Utrecht, The Netherlands; 2 Laboratory of Cognitive Science and Immersive Virtual Reality, Department of Psychology, Second University of Naples, Caserta, Italy; 3 Faculty of Social and Behavioral Sciences, Leiden University, Leiden, The Netherlands

**Keywords:** Verbal response, Egocentric/allocentric frames of reference, Categorical/coordinate spatial relations, Ventral stream

## Abstract

The aim of this study was to explore how people use egocentric (i.e., with respect to their body) and allocentric (i.e., with respect to another element in the environment) references in combination with coordinate (metric) or categorical (abstract) spatial information to identify a target element. Participants were asked to memorize triads of 3D objects or 2D figures, and immediately or after a delay of 5 s, they had to verbally indicate what was the object/figure: (1) closest/farthest to them (*egocentric coordinate task*); (2) on their right/left (*egocentric categorical task*); (3) closest/farthest to another object/figure (*allocentric coordinate task*); (4) on the right/left of another object/figure (*allocentric categorical task*). Results showed that the use of 2D figures favored categorical judgments over the coordinate ones with either an egocentric or an allocentric reference frame, whereas the use of 3D objects specifically favored egocentric coordinate judgments rather than the allocentric ones. Furthermore, egocentric judgments were more accurate than allocentric judgments when the response was Immediate rather than delayed and 3D objects rather than 2D figures were used. This pattern of results is discussed in the light of the functional roles attributed to the frames of reference and spatial relations by relevant theories of visuospatial processing.

## Introduction

Encoding and representing spatial information is a fundamental prerequisite for many daily life activities. When you are looking for the car keys, you need to remember the exact position you left them the last time, whereas if you have to describe a scene or recognize a place, you need to remember, for example, what was the building on the right (or left) of another or if a certain building was close (or far) to you or to another one. These examples suggest that people are able to represent spatial information according to two frames of reference (FoR): egocentric (i.e., observer based) and allocentric (i.e., scene based). Furthermore, they also underscore that spatial relations (SR) can be coordinate, that is, fine-grained metric information that allows for exact distance comparisons, or categorical, that is, more abstract such as right/left or above/below.

The distinction between categorical and coordinate spatial relations is supported by computer simulation, behavioral, neurofunctional, and neuropsychological studies (see Jager and Postma 2003; Postma and Laeng 2006; Van der Ham et al. 2014). In particular, it has been shown that categorical and coordinate representations are subserved by separate neural circuits in the left hemisphere and in the right hemisphere, respectively (Hellige and Michimata 1989; Kosslyn et al. 1989; Laeng 1994, 2006; Trojano et al. 2006; van Asselen et al. 2006; Van der Ham et al. 2012a, b, 2013a, b). In the same way, behavioral data support the existence of egocentric and allocentric frames of reference (Presson and Hazelrigg 1984; Presson et al. 1989; McNamara et al. 2003; Kelly et al. 2007; Iachini et al. 2009a), and many neurofunctional studies have shown that the two frames of reference would engage distinct neural networks, with a parietal-premotor network supporting both egocentric (more right-sided) and allocentric representations and a further involvement of ventromedial occipito-temporal cortex for allocentric representations (Committeri et al. 2004; Galati et al. 2000; Iachini et al. 2009b; Ruggiero et al. 2014; Vallar et al. 1999; Zaehle et al. 2007; Neggers et al. 2006; Chen et al. 2014).

It has been suggested that categorical and coordinate spatial relations have a functional role similar to that attributed to the allocentric and egocentric frames of reference, respectively (Kosslyn 1987, 2006; Milner and Goodale 1995, 2008). According to the “two-visual stream hypothesis” proposed by Milner and Goodale (1995, 2008) and Goodale 2014), allocentric and egocentric frames of reference have a clear and distinct functional role within perceptual- and action-oriented tasks. Specifically, the vision-for-action subsystem (dorsal stream) would privilege egocentric frames of reference for controlling movements in space. Instead, the vision-for-perception subsystem (ventral stream) being related to visual consciousness and to memory systems would privilege allocentric frames of reference. Similarly, Kosslyn (1987) proposed that categorical information is more useful for object recognition, whereas coordinate spatial relations are more useful for accurately reaching elements in the space (object or places).

In a first study aimed at understanding the relationship between FoR and SR processing, Ruotolo et al. (2011a), but see also Ruotolo et al. (2011b), asked participants to judge whether two 2-dimensional vertical bars were on the same side (categorical task) or at the same distance (coordinate task) with respect to their body midline (egocentric reference) or with respect to an horizontal bar (allocentric reference). Results showed that categorical judgments with respect to the allocentric reference were more accurate than all others. More recently, the influence of the characteristics of the stimuli, that is, 3D objects versus 2D figures, and the temporal parameters of the response, that is, Immediate versus delayed, on the combination between FoR and SR was studied with the use of a visuo-motor task (Ruotolo et al. 2015). In this task, participants had to indicate, by reaching and touching, the position previously occupied by the stimulus closest/farthest to them (egocentric coordinate) or to another stimulus (allocentric coordinate), or if a stimulus was on the right/left side with respect to them (egocentric categorical) or to another stimulus (allocentric categorical). Results showed an advantage of egocentric over allocentric coordinate judgments independently from the kind of stimuli used and the temporal parameters of the response, whereas no difference appeared between egocentric and allocentric categorical judgments when 2D stimuli were used and a Delayed response was required.

In sum, the results from these two studies seem to suggest that a task boosting motor components with the use of manipulable objects and an Immediate motor response (i.e., reaching and touching) (Ruotolo et al. 2015) would favor the combination of coordinate spatial relations and egocentric reference frames; instead, a task with a visuo-perceptual response (i.e., judging spatial locations by verbal response or response keys pressing) and non-manipulable figures (Ruotolo et al. 2011a, b) would favor more abstract and relational spatial components, such as categorical and allocentric. However, these conclusions are drawn from studies that differ not only in the response modality (visuo-motor vs. visuo-perceptual), but also with respect to the stimuli and procedural details. For example, in Ruotolo et al. (2011a), only non-manipulable stimuli and an Immediate visuo-perceptual response were used. More importantly, some evidence, labeled by Foley et al. (2015) as the “perspectival accounts of visual experience,” argues against the possibility that the mere use of visuo-perceptual tasks would favor allocentric rather than egocentric spatial representations due to the fundamentally egocentric nature of visual experience.

Therefore, in order to clarify the relationship between FoR and SR processing in a visuo-perceptual task (i.e., not requiring pointing or reaching a spatial location), we decided to replicate our previous study (2015) in which both the kind of stimulus and the temporal parameters were manipulated, but now with a new response modality: visuo-perceptual judgment instead of visuo-motor pointing. Our spatial task explicitly requires the encoding of distances (coordinate) or relations (categorical) with respect to the participant’s body (egocentric) or to an external object (allocentric). This kind of experimental paradigm has already been used to assess spatial memory in healthy adults (Iachini and Ruggiero 2006), brain damaged patients (Barca et al. 2010; Ruggiero et al. 2014), blind people (Ruggiero et al. 2009a, 2012; Iachini et al. 2014a), children with cerebral palsy (Barca et al. 2012), in a fMRI study (Committeri et al. 2004), and has proved its efficacy in inducing a specific involvement of spatial frames of reference. In the current study, one group of participants was required to learn the position of three geometrical objects (“3D” condition), whereas another group learned the position of three 2-dimensional geometric figures (“2D” condition). After removing the stimuli, participants were asked to verbally indicate what was the object/figure closest/farthest to them (*egocentric coordinate task*) or to another object/figure (*allocentric coordinate task*) and what was the object/figure on their right/left (*egocentric categorical task*) or on the right/left of another object/figure (*allocentric categorical task*). In both 3D and 2D conditions, participants were divided into two subgroups: A subgroup was requested to give the answer immediately after (i.e., after 1.5 s) stimuli removal (“Immediate” response), whereas the other subgroup to give the answer after 5-s stimuli had been removed (“Delayed” response).

According to the “perspectival account,” a general advantage of egocentric rather than allocentric organization of spatial information should emerge even if this is a visuo-perceptual and not a visuo-motor task. However, on the basis of our previous study the differences between allocentric and egocentric judgments should depend on the kind of required spatial relations, the characteristics of the stimuli, and the temporal parameters of the response. Specifically, since the use of 3D manipulable stimuli with an Immediate response is supposed to stress the dorsal stream of the brain (Ellis and Tucker 2000; Tucker and Ellis 1998, 2011, 2004; Iachini et al. 2008, 2014b), egocentric representations of coordinate relations should be favored over allocentric ones. Instead, a Delayed response with 2D non-manipulable figures should improve allocentric (Ball et al. 2009; Chen et al. 2011) and categorical spatial relations (van der Ham et al. 2007).

To verify these predictions, an experiment with four experimental conditions was carried out.

## Methods

### Participants

Ninety-six students from the Second University of Naples and Utrecht University participated in the experiment in exchange for course credit or a small amount of money. They were randomly assigned to one of the four experimental conditions but matched on the basis of sex and age: “Immediate-3D” condition (12 men and 12 women, mean age = 22.20, SD = 1.80); “Delayed-3D” condition (12 men and 12 women, mean age = 23.40, SD = 2.60); “Immediate-2D” condition (12 men and 12 women, mean age = 21.50, SD = 1.60); “Delayed-2D” condition (12 men and 12 women, mean age = 18.40, SD = 2.45). All participants were right handed and had normal or corrected to normal vision. Recruitment and testing were in conformity with the requirements of the Ethical Committee of the Second University of Naples, of the Ethical Committee of the Faculty of Social and Behavioral Sciences of Utrecht University, and of the 2013 Declaration of Helsinki.

Informed consent was obtained from all participants.

### Setting and materials

The experiment was carried out in a soundproofed, comfortable room. Participants sat on a straight-back chair placed centrally at 30 cm from the edge of a small desk measuring 50 cm (width) × 35 cm (length).

Stimuli and setting were the same as used by Ruotolo et al. (2015 (see also Iachini and Ruggiero 2006; Iachini et al. 2014a; Ruggiero et al. 2014). The stimuli comprised easily nameable and well-known 3D geometrical objects such as Pyramid, Parallelepiped, Cone, Cube, Sphere, and Cylinder and the corresponding 2D geometrical figures (i.e., Pyramid = Triangle; Parallelepiped = Rectangle; Cube = Square; Cone = Circle; Sphere = Circle; Cylinder = Circle). As regards 3D objects, they could have two sizes: big (8 cm × 8 cm, except Parallelepiped and Cylinder: 8 cm × 11 cm) and small (6 cm × 6 cm, except Parallelepiped and Cylinder: 6 cm × 9 cm). They differed in color: dark, medium, and light gray. The combination of objects, size, and color was such that 18 objects were obtained (e.g., the Cone could be big-dark), subdivided into two series [(A) Pyramid, Parallelepiped, and Cone; and (B) Cube, Sphere, and Cylinder]. Still, each series was subdivided into three triads. Each triad had a target object (T) that is the object with respect to which the allocentric judgments were given. Each triad was arranged on the desk on a plasterboard panel (50 cm × 30 cm × 2 cm) according to the following criteria: (1) inter-objects metric distances had to be easily distinguishable; (2) the metric distances were established in such a way that the amount of metric difficulty was the same for egocentric and allocentric judgments. The metric difficulty was related to the amount of distance between stimuli. A total of 24 different triads were obtained, so each spatial judgment was given on a different configuration of objects/figures. The arrangement of the materials was based on pilot studies presented in previous reports (Iachini and Ruggiero 2006; Ruotolo et al. 2015). To guarantee that all triads were presented in the same way for all participants, each triad was presented by means of a panel with the same size of the desk placed in front of participants. On this panel, the shape forming the basis of each object was engraved and the corresponding object was placed there. As regards 2D stimuli, the same logic was applied for the construction of the triads and the figures were drawn with a black pencil on panels of the same dimensions of those used for the objects.

### Procedure

 Participants were first given written instructions about the procedure. Next, there was a training session using three common objects (e.g., a glass, a cup, and a small box). Afterward, all experimental stimuli were presented and participants had to name them. In this way, difficulties due to naming problems could be excluded. Finally, the experiment started.

#### Learning phase

**Fig. 1:**
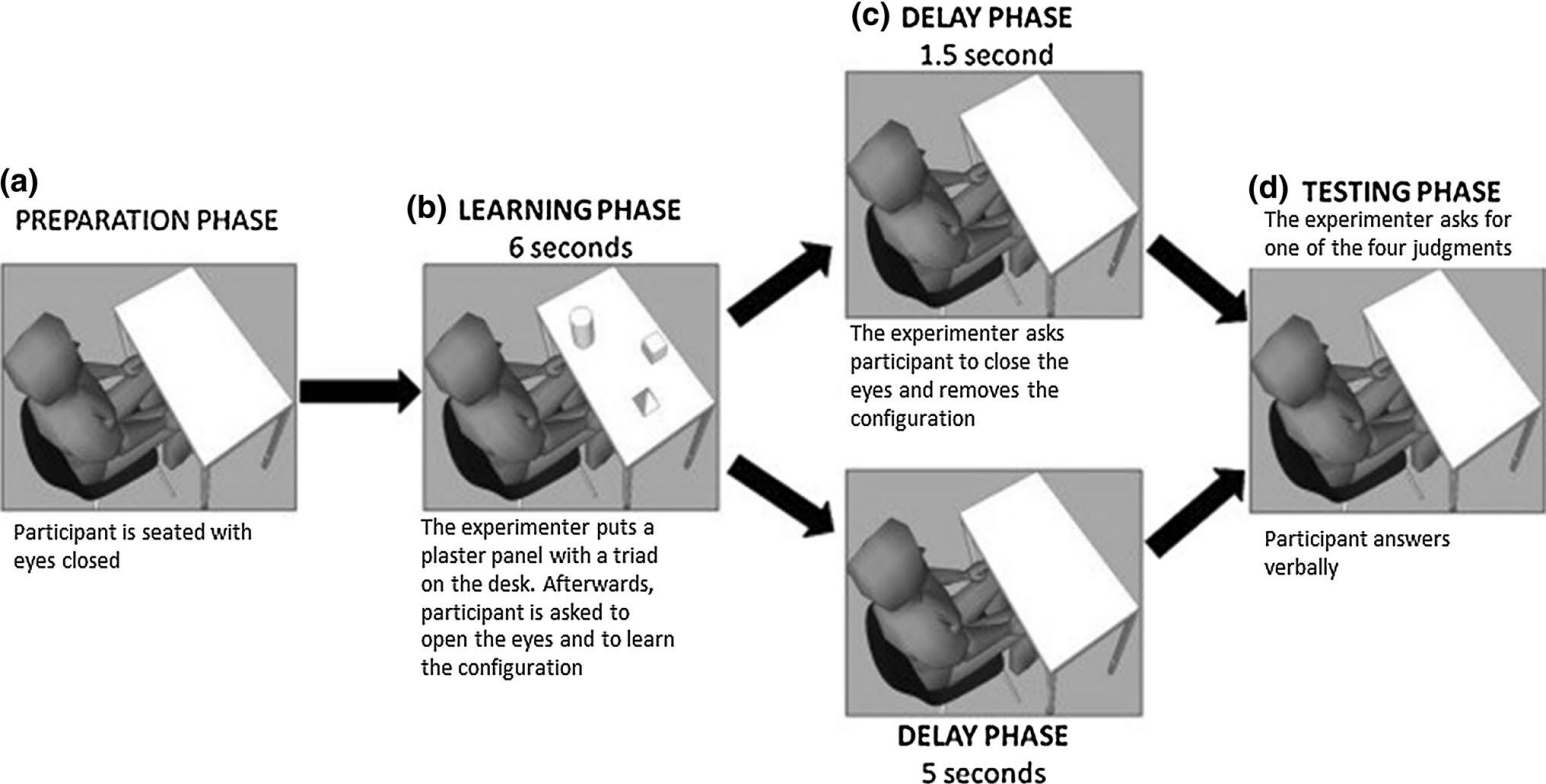
Schematic overview of the learning and testing phase. **a** Participant is seated at the desk with the eyes closed and the experimenter helps him\her to align his\her body midline with the center of the desk; **b** the experimenter puts a triad on the desk and asks participant to open the eyes and to learn the objects or the figures (not shown here) and their position; **c** according to the assigned experimental condition, participant has to wait 1.5 or 5 s to receive the instructions about the kind of spatial judgment requested (Ego-Coor or Ego-Cat or Allo-Coor or Allo-Cat). During this time, participant is with eyes closed and the triad is removed; **d** finally, the experimenter asks for the spatial judgment

While participants had their eyes closed the experimenter posited the panel on the desk. Afterward, participants were asked to open their eyes and memorize (6 s) the three objects/figures and their positions. Learning time was monitored with a stopwatch. After the 6-s learning, the experimenter asked participants to close their eyes while removing the panel with the objects/figures from the desk. Next, the testing phase began after 1.5 s or after 5 s from stimuli removal according to the experimental condition (Immediate vs. Delayed condition) (see Fig. 1).

#### Testing phase

After memorizing a triad, participants had to verbally answer to one of four kinds of questions: (a) egocentric coordinate (Ego-Coor), “What was the object/figure closest (or farthest) to you?”; (b) egocentric categorical (Ego-Cat), “What was the object/figure on your right (or left)?”; (c) allocentric coordinate (Allo-Coor), “What was the object/figure closest (or farthest) to the target (e.g., Cylinder/Circle)?”; and (d) allocentric categorical (Allo-Cat), “What was the object/figure on the right (or left) of the target (e.g., Cube/Square)?”. Importantly, the experimenter asked for the spatial judgments by using only two words: “Closest-YOU” or “Farthest-YOU” for Ego-Coor judgment, “Closest-OBJECT X” or “Farthest-OBJECT X” for Allo-Coor judgment, “Right-YOU” or “Left-YOU” for Ego-Cat judgments, “Right-OBJECT X” or “Left-OBJECT X” for Allo-Cat judgments. These instructions were explained in the training session and allowed to ask for spatial judgments in a very short delay (about 700 ms for each instruction).

In the “Immediate condition,” instructions were given after 1.5 s from stimuli disappearance, whereas in the “Delayed condition” instructions were given after 5 s. It is important to notice that participants did not know in advance what of the four spatial judgments they would have been requested.

Immediately after the experimenter gave the instructions for the spatial judgment, a stopwatch was activated and participants gave the response. Afterward, the experimenter manually reported both the response time and the answer on a sheet.

A total of 24 responses were given (six responses for each kind of judgment). The order of presentation of the questions was first randomized and then balanced across participants.

### Data analysis

An ANOVA for mixed design was carried out on accuracy (0/1, score range = 0–6 for each spatial combination; the mean accuracy per participant was calculated) with FoR (egocentric vs. allocentric) and SR (coordinate vs. categorical) as within variables and Delay (1.5 vs. 5 s) and Stimuli (2D vs. 3D) as between factors. The Scheffé test was used to analyze post hoc effects. The magnitude of effect sizes was expressed by η_p_^2^. Finally, confidence intervals (95 %) for the means were indicated.

## Results

Results showed a main effect of FoR due to egocentric judgments (*M* = .87; SD = .15; 95 % CI [.84, .89]) being more accurate than allocentric ones (*M* = .79; SD = .17; 95 % CI [.76, .81]) (*F*(1, 92) = 30.07, *p* < .001, *η*_p_^2^ = .25), a main effect of SR due to categorical judgments (*M* = .86; SD = .15; 95 % CI [.84, .89]) being better than coordinate ones (*M* = .79; SD = .17; 95 % CI [.76, .81]) (*F*(1, 92) = 20.68, *p* < .001, *η*_p_^2^ = .18), and a main effect of Delay due to judgments delayed by 5 s (*M* = .85; SD = .16; 95 % CI [.82, .87]) being more accurate than the immediate ones (*M* = .81; SD = .17; 95 % CI [.78, .83]) (*F*(1, 92) = 6.81, *p* < .05, *η*_p_^2^ = .07). Furthermore, a two-way interaction between Stimuli and Delay also appeared: *F*(1, 92) = 11.63, *p* < .001, *η*_p_^2^ = .11. The post hoc test showed that it was due to the delayed judgments toward 3D stimuli (*M* = .88; SD = .14; 95 % CI [.86, .90]) being more accurate than the immediate ones (*M* = .78; SD = .19; 95 % CI [.74, .82]) toward the same kind of stimuli (*p* = .0008). No significant difference appeared for the 2D stimuli (immediate: *M* = .83, SD = .15, 95 % CI [.80, .86]; Delayed: *M* = .82, SD = .18, 95 % CI [.78, .86]; *p* = .96). This interaction was specified by a three-way interaction between Stimuli, Delay, and FoR: *F* (1, 92) = 5.1289, *p* = .026, *η*_p_^2^ = .05 (see Table 1).

**Table 1:** Mean accuracy, standard deviations (SD), and confidence interval (CI) for egocentric and allocentric judgments, 2D and 3D stimuli and immediate versus delayed answer

	Immediate				Delayed			
	EGO 2D	EGO 3D	ALLO 2D	ALLO 3D	EGO 2D	EGO 3D	ALLO 2D	ALLO 3D
Mean	0.833	0.840	0.825	0.725	0.871	0.924	0.760	0.837
SD	0.102	0.122	0.077	0.119	0.125	0.092	0.119	0.090
*95* *% CI*								
Upper bound	0.875	0.891	0.858	0.775	0.925	0.962	0.811	0.875
Lower bound	0.790	0.788	0.793	0.675	0.819	0.885	0.710	0.799

The post hoc test showed that egocentric judgments (*M* = .84; SD = .15) were better than allocentric ones (*M* = .72; SD = .20) when the judgments were immediate and 3D stimuli were used (*p* = .04). Egocentric judgments (*M* = .87; SD = .15) were better than allocentric ones (*M* = .76; SD = .16) also when the judgments were delayed and 2D stimuli were used (*p* = .055). Furthermore, a tendency appeared due to allocentric judgments with 3D stimuli being better after 5 s (*M* = .84; SD = .20) than immediately (*M* = .72; SD = .15) (*p* = .075). Instead, no difference appeared between egocentric and allocentric judgments when 3D stimuli were combined with a delayed answer (*p* = .28) and when 2D stimuli were combined with an immediate answer (*p* = 1).

**Fig. 2:**
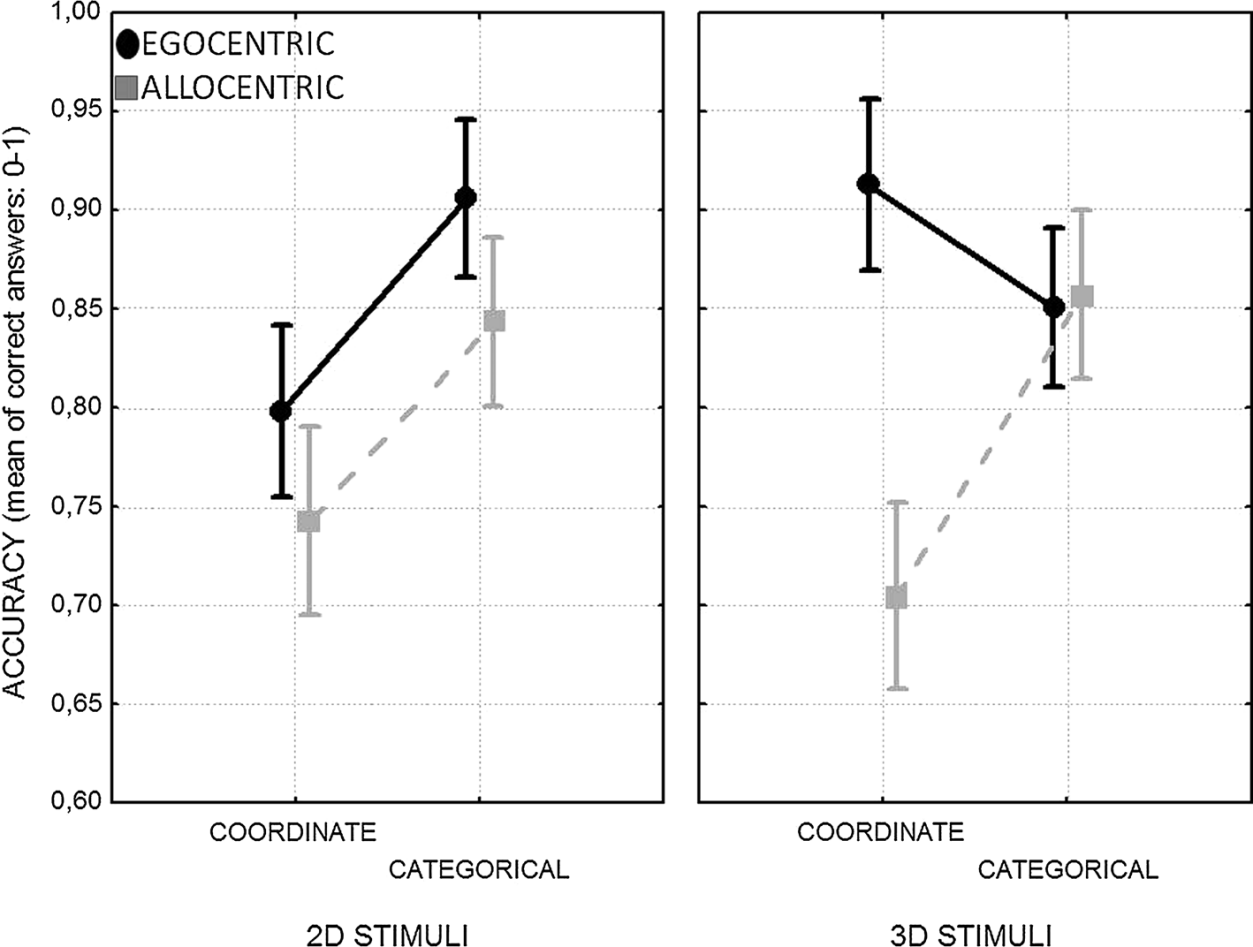
Graph on the *left* shows the mean accuracy of coordinate and categorical judgments as a function of the egocentric and allocentric reference frames with 2D stimuli. Instead, the graph on the *right* shows the mean accuracy for the same judgments with 3D stimuli

Finally, a two-way interaction between FoR and SR was found: *F* (1, 92) = 12.95, *p* < .001, *η*_p_^2^ = .12. The post hoc test showed that it was due to allocentric coordinate judgments (*M* = .72; SD = .17; 95 % CI [.69, .76]) being worse than all others (egocentric coordinate: *M* = .85, SD = .16, 95 % CI [.82, .89]; egocentric categorical: *M* = .88, SD = .14, 95 % CI [.85, .91]; allocentric categorical: *M* = .85; SD = .16, 95 % CI [.82, .88]) (*p*_s_ = .000001). However, this interaction was modulated by the kind of stimuli used: *F* (1, 92) = 14.723, *p* < .001, *η*_p_^2^ = .14. The post hoc test showed that the three-way interaction was due to egocentric categorical judgments being better than egocentric coordinate ones when 2D (*p* = .065) and not 3D stimuli (*p* = .65) were used. Instead, egocentric coordinate judgments were more accurate with 3D than with 2D stimuli (*p* = .054). Furthermore, with 3D stimuli, allocentric coordinate judgments were worse than all other judgments (at least *p* < .001), whereas with 2D stimuli no difference appeared between egocentric and allocentric judgments for both categorical (*p* = .69) and coordinate spatial relations (*p* = .81) (see Table 2; Fig. 2). No other significant effect was found (at least *p* > .20).

**Table 2:** Mean accuracy, standard deviations (SD), and confidence interval (CI) for each spatial judgment for 2D and 3D stimuli

	2D stimuli				3D stimuli			
	Ego-Coor	Ego-Cat	Allo-Coor	Allo-Cat	EGO-Coor	Ego-Cat	Allo-Coor	Allo-Cat
Mean	0.798	0.906	0.743	0.843	0.913	0.851	0.705	0.857
SD	0.18	0.12	0.17	0.14	0.11	0.16	0.16	0.17
*95* *% CI*								
Upper bound	0.852	0.940	0.791	0.884	0.944	0.899	0.753	0.908
Lower bound	0.745	0.871	0.694	0.803	0.881	0.803	0.657	0.806

## Discussion

The aim of this study was to verify whether egocentric and allocentric representations were influenced by the kind of spatial relations required (coordinate vs. categorical), the characteristics of stimuli (3D vs. 2D), and the temporal parameters of the response (Immediate vs. Delayed response). In line with our hypotheses, the results showed that the way people represent spatial information is influenced by the characteristics of the task at hand.

As regards spatial relations, an advantage of egocentric over allocentric coordinate judgments was found, whereas no significant difference appeared between egocentric and allocentric categorical judgments. Focusing on categorical relations, we may argue that the lack of difference cannot be attributed to categorical judgments being overall easier than coordinate ones since the two kinds of judgments had the same level of accuracy when combined with an egocentric frame. Instead, results showed a specific difficulty in representing coordinate spatial relations in an allocentric way (for a discussion about the relative difficulty of coordinate over categorical spatial relations, see: Kosslyn 1994; Bruyer et al. 1997; Parrot et al. 1999; Dent 2009; Jager and Postma 2003). This difficulty was particularly clear with 3D stimuli.

As regards the characteristics of the stimuli, the three-way interaction confirmed that this factor modulated the relationship between frames of reference and spatial relations. Specifically, allocentric coordinate judgments with 3D stimuli were worse than all other judgments, whereas egocentric coordinate judgments with 3D stimuli were better than with 2D stimuli. Furthermore, the egocentric categorical combination was better than the egocentric coordinate combination when 2D but not 3D stimuli were used, whereas allocentric categorical judgments were always better than coordinate ones. In sum, the use of 3D objects clearly favored egocentric coordinate judgments over allocentric ones, whereas the use of 2D figures favored categorical judgments. In contrast, such a clear difference between egocentric and allocentric judgments did not appear with 2D stimuli, for either categorical or coordinate judgments. However, these last results should be interpreted with caution. Even if the post hoc did not show differences between egocentric and allocentric coordinate judgments on the one side, and egocentric and allocentric categorical judgments on the other, their confidence intervals were only partially overlapping and this offered a weak support to the null hypothesis (i.e., absence of a difference for these comparisons).

As regards the delay, results showed that participants were overall more accurate in giving spatial judgments when a delay of 5 s was introduced between learning and testing with respect to the immediate answer condition. However, this advantage was modulated by the characteristics of the stimuli and the frames of reference. Specifically, egocentric judgments were better than allocentric ones when either an immediate answer was combined with 3D objects or a delayed answer was combined with 2D figures. Moreover, egocentric judgments were only slightly better than allocentric judgments with a delayed answer and 3D objects, whereas no difference between egocentric and allocentric judgments appeared with an immediate answer and 2D figures.

It is interesting to notice that this pattern of results shows some differences with respect to the one that emerged from our previous study (Ruotolo et al. 2015). Results from the visuo-motor study, in which participants were required to reach for and touch the position occupied by a target object, showed that egocentric coordinate judgments were better than allocentric ones independently from the kind of stimuli (3D vs. 2D) and the temporal parameters of the response (Immediate vs. delayed). Furthermore, an advantage of egocentric over allocentric categorical judgments also appeared, but only for the immediate judgments toward 3D stimuli. Finally, the 5-s delay made allocentric categorical judgments faster and more accurate. The comparison between the two patterns of data suggests that the use of a visuo-perceptual task combined with 2D stimuli reduces the relevance of coordinate, metric, spatial relations by favoring the processing of categorical, abstract, spatial relations. Even the request of an Immediate response does not negatively affect allocentric categorical judgments, as it happened with the visuo-motor response.

What are the implications of the current results? They partially support the “perspectival account” of the visual experience (Foley et al. 2015) and the role attributed to the frames of reference in the perception–action model (Milner and Goodale 1995, 2008). According to the “perspectival account,” an egocentric over allocentric advantage should emerge irrespective of the kind of task, whereas according to Milner and Goodale, a visuo-perceptual task should favor allocentric representations. The results from this study show that a clear egocentric over allocentric advantage emerges only in specific circumstances, that is, when coordinate spatial relations are processed and an Immediate response to 3D stimuli is required. This implies that the way people process and represent spatial information could be modulated not only by the nature of the response (i.e., visuo-motor or visuo-perceptual), as suggested by Milner and Goodale, but also by the stimulus characteristics and temporal parameters of the response. In other words, an egocentric over allocentric advantage can also appear with a visuo-perceptual task if other components of the task, such as presence of 3D manipulable objects or Immediate response, boost a motor encoding of spatial properties. On the opposite side, a visuo-motor task can be solved by using allocentric representations particularly when the answer is delayed, that is, memory based, and more abstract stimuli are used.

However, the fact that the four-way interaction among frames of reference, spatial relations, delay, and characteristics of the stimuli was not significant could suggest that the use of a spatial representation rather than another does not strictly reflect the “quantity” of visuo-motor or visuo-perceptual properties of the task. If this were the case, results should have shown an advantage of allocentric judgments over egocentric ones when categorical spatial relations had to be recovered from memory and 2D figures were used. Instead, we have still found an advantage of egocentric judgments with a delayed answer and 2D figures. This induces to some considerations about the properties of spatial representations.

First, two kinds of egocentric representations could be distinguished: a short-duration one that is used to represent precise spatial information useful during the online control of movements; a long-lasting and one that is used to organize in memory coordinate and categorical spatial information and therefore is useful for visuo-perceptual task or for planning a future movement. This is also in line with much evidence coming from studies about spatial memory (e.g., Shelton and McNamara 2001; Wang and Spelke 2002; Kelly et al. 2007), showing that object locations are encoded mainly in an egocentric manner even if allocentric representations would exist in parallel (Burgess 2006). Instead, in their perception–action model Milner and Goodale (1995, 2008) only refer to the short-term egocentric representations, while highlighting the role of allocentric representation in memory-based tasks. On this basis, it is hard to think of egocentric and allocentric representation as being encapsulated within a strict division of the labor between dorsal and ventral streams, instead it seems more plausible that different kinds of spatial representations (i.e., at least four: egocentric coordinate, egocentric categorical, allocentric coordinate, and allocentric categorical) can flexibly contribute to memory-based, visuo-perceptual, or visuo-motor tasks. However, future experiments in which visuo-motor and visuo-perceptual responses can be directly compared as well as neuroimaging studies exploring the neural correlates of these different kinds of spatial representations are needed to give further support to these speculations. Furthermore, it will be necessary to verify whether different kinds of coordinate (e.g., distance estimation: Böök and Gärling, 1980; Étienne, Maurer, and Séguinot, 1996; pointing a location from a different heading: Mou, McNamara, Valiquette, and Rump, 2004; Loomis and Knapp, 2003) and categorical judgments (Bauman et al., 2012) have a different influence on the use of egocentric and allocentric frame of reference during navigational tasks.

Second, our results suggest that allocentric representations are more difficult to build up compared with egocentric ones especially when coordinate rather than categorical relations and 3D objects are involved. According to Millar (1994), this difficulty could be ascribed to the dominance of the egocentric perspective in our bodily interaction with the environment. As a consequence, more processes would be needed to detach from this perspective in order to represent spatial information allocentrically. However, results from this study show that the egocentric advantage suggested by Millar (1994) cannot be generalized.

Finally, we should consider the possibility that the egocentric advantage we found was due to some artifacts that facilitated the egocentric performance to the detriment of the allocentric one. For example, in our task participants remained seated in the same place throughout the experiment. As a consequence, they could rely on a clear and stable egocentric frame of reference (i.e., their body). On contrast, allocentric judgments were requested with respect to one of three possible stimuli of each configuration. According to Waller et al. (2002) and Kelly et al. (2007), the sensorimotor awareness of a stable orientation and the stability of some environmental landmarks (e.g., the walls of the room) could favor participants’ encoding of spatial information in egocentric rather than allocentric terms. However, on the basis of our previous control experiments we could discard this possible confound. For example, in Ruggiero et al. (2009b) the egocentric reference frame was made variable by changing the learning position: Six triads of stimuli were placed on six different sections of a desk and participants had to study them by sitting in front of the various sections. Instead, in Ruotolo et al. (2015), in a first control study the position of the participant between encoding (learning) and retrieval (testing) was changed, thereby making unstable the egocentric reference frame. Four positions within the room were devised (P1, P2, P3, and P4) from which participants had a different view of the room. So, if participant learned the triad of objects in P1, then he/she was asked to move to P2 (or P3 or P4) for the testing. Differently from Kelly et al. (2007), we could not allow participants to move to a different room for the testing phase because of time constraints (5 s of delay between learning and testing). In a second control experiment, we made the allocentric reference point as clear and stable as the egocentric reference frame. A black plastic box was posited on the side of the desk opposite to the participants, and allocentric judgments were always given with respect to this reference point. In brief, the pattern of results from these three control experiments confirmed the egocentric over allocentric advantage and discarded the possibility that it was due to an artifactual facilitation.

## Conclusions

In conclusion, the results suggest that several characteristics of the task, such as the temporal parameters of the responses, the characteristics of the stimuli as well as the kind of spatial relation required, can flexibly influence the egocentric and/or allocentric encoding of spatial information. Specifically, the comparison with the results from our previous study (Ruotolo et al. 2015) seems to suggest that the use of a visuo-perceptual, rather than visuo-motor, task facilitates the representation of more abstract categorical spatial relations and reduced, in some circumstances, the advantage of egocentric over allocentric judgments.
